# Book Review: The Neurocognition of Translation and Interpreting

**DOI:** 10.3389/fpsyg.2021.715226

**Published:** 2021-07-02

**Authors:** Yi Shan, Ling Li

**Affiliations:** ^1^School of Foreign Studies, Nantong University, Nantong, China; ^2^College of Foreign Languages, Fujian Normal University, Fuzhou, China; ^3^Shandong Academy of Social Sciences (SASS), Jinan, China

**Keywords:** neurocognition, translation, interpreting, book review, Adolfo M. García, John Benjamins

Since the advent of Translation and Interpreting Studies (TIS), researchers have been committing themselves to it from a multitude of interdisciplinary perspectives. However, “these approaches seldom draw on brain-informed data and rely predominantly on linguistic and behavioral results” (p. XI). Thus, the neural basis of interlingual transfer still remains one of the “known unknowns” (Tymoczko, 2012: 83) in TIS. In this context, inspired by 4EA cognitive view (embedded, extended, embodied, enacted, and affective) (Muñoz Martín, 2017; Risku, 2017) on translation and interpreting (TI), Adolfo M. García has taken a quite bold step forward to look at TI in the brain in *The Neurocognition of Translation and Interpreting*. The intended readers are scholars in the fields of cognitive TIS, bilingualism, and neurolinguistics, as well as the TI community, including teachers, students, and practitioners.

As a groundbreaking work, this book describes neurocognitive research into TI, aiming at achieving five objectives: (i) introducing neurocognitive research on interlingual reformulation (IR) in relation to other cognitive approaches; (ii) describing the methodologies adopted in the literature; (iii) presenting key notions of neurology, the neural basis of language, and the neurocognitive particularities of bilingualism, to examine findings about IR; (iv) compiling, organizing, and interpreting neuropsychological, neuroscientific, and behavioral evidence on highly prominent topics for TIS; and (v) discussing the present and future of TI. In addition to a preface and an introduction, this research piece comprises eight chapters, which can roughly be divided into three parts, to, respectively, deal with the theoretical foundations (chapters 1–3), four highly prominent topics for TIS (chapters 4–7), and the reflection on and prospect of neurocognitive TIS (chapter 8).

Part I begins with chapter 1 in which Adolfo M. García locates neural approaches to IR within the broad purview of cognitive TIS. Chapter 2 gives a presentation of the methodological toolkit employed for the examination of the neurocognitive respects of IR. Chapter 3, mainly directed at readers unfamiliar with neuroscience and neurolinguistics, gives an account of basic concepts and interpretive constraints for neurocognitive characterizations of IR. The four chapters in the second part of this volume systematize neuropsychological, neuroscientific, and behavioral evidence on four highly prominent topics for TIS, including translation disorders of IR in brain-damaged bilinguals, brain-based research on directionality, the construct of translation units, and the necessity of neurocognitive adaptations. Part III takes a critical look at the present and future of TIS, by identifying the accomplishments, strengths, weaknesses, and requirements at methodological, theoretical, and institutional facets.

The main contribution and distinct feature of this book is as follows: Taking the neurocognitive research of interlingual transfer as the axis, and based on fine-grained evidence from such research, this volume puts forth and demonstrates three views on the neurocognitive research on TI, including the “known view,” “complementarity view,” and “cooperative view.” García points out that with the existing neuroimaging technology, electrophysiological technology, and brain stimulation technology, the research on TI can already explain the neurocognitive basis of interlingual transfer in TI from the aspects of brain anatomy, brain function, brain plasticity, and brain behavior. His seamless in-depth synthesis of the core concepts and major discoveries concerning TIS succeeds in turning the “known unknown” into the “unknown known” to some extent. Besides, García believes that the neurocognitive research into TI is a useful supplement to its non-neurocognitive counterpart. These two research approaches are in a complementary and positive co-construction relationship, jointly promoting the development of cognitive research on TI. Although the development of cognitive research into TI has been polarized into the micro in-depth neurocognitive investigation and the macro sociocognitive expansion (Muñoz Martín, 2017), these two approaches are complementary, with the former being the underlying bio-psychological basis of the latter. Moreover, García proposes that researchers of TI and neurocognitive science should cooperate with each other to promote the development of TIS.

Another contribution of this book lies in its universal hypothesis or theoretical construction of neurocognitive research on TI. It points out that four factors, including translation processing pathways, different translation directions, source-language units to be processed, and the interpreter's expertise, are indispensable for the empirical research and the theoretical construction of neurocognitive TIS. Otherwise, it is difficult to fully reveal the cognitive process of interlingual transfer in the brain of interpreters and translators. It also fully demonstrates the role of the aforementioned four factors in the construction of the neurocognitive theory of TI, and systematically describes and integrates this theory based on interlingual transfer.

On the whole, this volume is authoritative, systematic, and pioneering. “Written by a leading neuroscientist and T&I researcher, García's book raises neurocognitive work in the field to new, impressive heights. To anyone interested in the topic, this volume will remain the standard reference work.” (García, 2019: back cover) It is justifiably the first work designed to systematically integrate the findings in the neurocognitive research on TI and systematically construct the neurocognitive theory of TI. This work is pioneering, in terms of calling for the need to embrace a neurocognitive approach to study TI. Without a shadow of doubt, looking at cognitive rhythms in the translator's and interpreter's brain definitely contributes to a better understanding of TI as activities embedded in the brain.

## Author Contributions

YS was mainly responsible for writing the first draft of this manuscript. LL proofread it and modified its format. All authors contributed to the article and approved the submitted version.

## Conflict of Interest

The authors declare that the research was conducted in the absence of any commercial or financial relationships that could be construed as a potential conflict of interest.

## References

[B1] García A. M. (2019). The Neurocognition of Translation and Interpreting. Amsterdam; Philadelphia, PA: John Benjamins.

[B2] Muñoz Martín R. (2017). “Looking toward the future of cognitive translation studies,” in The Handbook of Translation and Cognition, eds Schwieter J. W. Ferreira A. (Hoboken, NJ: John Wiley & Sons, Inc.), 555–572.

[B3] Risku H. (2017). “Ethnographies of translation and situated cognition,” in The Handbook of Translation and Cognition, eds by Schwieter J. W. Ferreira A. (Hoboken, NJ: John Wiley & Sons, Inc.), 290–310.

[B4] Tymoczko A. (2012). The neuroscience of translation. Target 1, 83–102. 10.1075/target.24.1.06tym

